# Supplementary material for: Understanding satisfaction and dissatisfaction of patients with telemedicine during the COVID-19 pandemic: An exploratory qualitative study in primary care
Source: PLoS One. 2023 Oct 17;18(10):e0293089. doi: 10.1371/journal.pone.0293089 (PMC10581451; doi:10.1371/journal.pone.0293089)
Supplement: Attachment — Submitted filename: PLOS one _reviewer comments.docx [file pone.0293089.s002.docx]

July 2023

**Dear Editor-in-chief,**

I would like to cordially thank you for giving me the chance to review the interesting manuscript entitled “Understanding satisfaction and dissatisfaction of patients with telemedicine during the COVID-19 pandemic: an exploratory qualitative study in primary care” which reported the results of a qualitative study conducted in Poland. The authors did a good job describing the research in general, however, I do have the following recommendation to improve the scientific soundness of the reported research:

**Abstract**

I do recommend elaborating more on the rationale of the study since the authors do have a window to do so. Please include more in the background section to highlight the importance of adoption of telemedicine during COVID-19. The authors are also advised to add details regarding the methodology of the study; it was so concise and missed major information.

**Introduction**

The authors discussed COVID-19 in depth and add enough details about telemedicine although it is the core of the study.

-	I do suggest focusing more on telemedicine; add definition, modalities of implementation, how well is Poland ready for adoption in regard to infrastructure and familiarity of both healthcare providers and patients.

-	Besides, I do suggest adding more about the rationale of the study; why is it important to study telemedicine and patient satisfaction dissatisfaction although the pandemic is now over? The authors need to convince the audience about their point of view.

**Methods/Results**

The methods section missed major pieces of information rendering the study heard to replicate.

-	I highly encourage the authors to adopt the Consolidated Criteria for Reporting Qualitative studies (COREQ) checklist or Standards for Reporting Qualitative Research (SRQR) Reporting checklist for qualitative study to add all missing information as it appears on the checklist in order to enhance the transparency of reporting of the qualitative study.

-	I was not able to sufficiently judge if the statistical analysis was appropriate given the missing information as explained above.

-	The authors did not clarify whether the raw data related to the research will be publicly available or restricted.

At the end, I would like to thank you again for giving me the chance to review this manuscript.

**Sincerely,**

Reviewer
