# Supplementary material for: Understanding satisfaction and dissatisfaction of patients with telemedicine during the COVID-19 pandemic: An exploratory qualitative study in primary care
Source: PLoS One. 2023 Oct 17;18(10):e0293089. doi: 10.1371/journal.pone.0293089 (PMC10581451; doi:10.1371/journal.pone.0293089)
Supplement: Attachment — Submitted filename: Response to reviewer2.docx [file pone.0293089.s003.docx]

Dear Reviewer,

We greatly appreciate the time you dedicated to reading our paper and providing us with your suggestions. The feedback you provided has proven invaluable in guiding us toward enhancing the quality of our manuscript.

Our next step is to attend to your comments.

A. Abstract

As per your suggestion, the methods section within the abstract succinctly summarizes the methodologies used in our qualitative research. Furthermore, we have added missing information to the background section.

B. Introduction

In the introduction section, we have delved extensively into the topic of telemedicine and its adoption within Poland. An extended introduction, containing additional information, is available on pages 3-7 of our manuscript.

C. Methods/Results

Acting upon your valuable suggestion, we have integrated the Consolidated Criteria for Reporting Qualitative Studies (COREQ) checklist.

This addition aims to incorporate any previously omitted information, aligning with the checklist's criteria to enhance the transparency of our qualitative study's reporting.

Substantial missing details have been seamlessly incorporated into the methods section.

All relevant data are within the manuscript and its Supporting Information files. Data cannot be shared publicity as participants did not give consent for their transcripts to be shared in the public domain. Data are available for selected researchers from the Medical University of Bialystok, who meet the criteria for access to confidential data. Requests for access to the underlying data may be directed to the Bioethics Committee of the Medical University of Bialystok [komisjabioetyczna@umb.edu.pl](mailto:komisjabioetyczna@umb.edu.pl)

Notable modifications have been highlighted in red throughout the text.

We truly hope the paper will be found suitable for publication.

Best regards,

Karolina Pogorzelska
